# Complete Genome Sequence of a Recombinant GII.P16-GII.4 Norovirus Detected in Kawasaki City, Japan, in 2016

**DOI:** 10.1128/genomeA.01099-16

**Published:** 2016-10-06

**Authors:** Yuki Matsushima, Tomomi Shimizu, Mariko Ishikawa, Ayako Komane, Nobuhiko Okabe, Akihide Ryo, Hirokazu Kimura, Kazuhiko Katayama, Hideaki Shimizu

**Affiliations:** a Division of Virology, Kawasaki City Institute for Public Health, Kanagawa, Japan; b Department of Microbiology, Yokohama City University School of Medicine, Kanagawa, Japan; c Infectious Disease Surveillance Center, National Institute of Infectious Diseases, Tokyo, Japan; d Department of Virology II, National Institute of Infectious Diseases, Tokyo, Japan

## Abstract

A recombinant norovirus, GII.P16-GII.4_Sydney2012, was first detected from nine patients with gastroenteritis in Kawasaki City, Japan, in 2016. The viral genome showed nucleotide sequence identities of 95.1% and 97.2% to the closest strains in the regions of 5′ terminus to ORF1 and ORF2 to 3′ terminus, respectively.

## GENOME ANNOUNCEMENT

Norovirus is a major causative pathogen of acute gastroenteritis in humans of all age groups (1). The virus is classified into genogroups I to VII (GI to GVII) (2). Patients with gastroenteritis are typically infected by noroviruses GI and GII, which have 9 and 22 genotypes, respectively. Given the emergence of recombinant strains, norovirus experts recommend that genotyping for worldwide surveillance use two proteins: the RNA-dependent RNA polymerase (RdRp) gene in open reading frame 1 (ORF1) and the capsid viral protein 1 (VP1) gene in ORF2 (3).

A novel recombinant norovirus was detected in the diarrheal feces of nine patients in an outbreak in Kawasaki City, Japan, in 2016. The strain was assigned to GII.P16-GII.4_Sydney2012 by the norovirus genotyping tool (4). One strain was used for complete genome sequencing. Primers for amplifying the 5′ terminus-ORF1 fragments of GII.P16-GII.4 strain were based on the published sequence of GII.P16 (KJ407074). The fragment of the ORF2-3′ terminus was amplified as described previously (5, 6). The complete genome of the strain was sequenced using primers based on the closely related sequences of GII.P16 (KJ407074) and GII.4_Sydney2012 (AB972482). The 5′-terminal sequence was analyzed using the 5′ Full RACE Core Set (TaKaRa, Shiga, Japan).

Except for its poly(A) tail, the complete genome of GII.P16-GII.4_Sydney2012 was 7,560 bp in length. Its GC compositions were 48.5% and 48.8% in the the 5′ terminus-ORF1 and ORF2-3′ terminus sequences, respectively. The strain had nucleotide sequence identities of 95.1% to the closest Hu/GII/RUS/2012/GII.P16/Smolensk/S12-31 strain (KF895841) in the 5′ terminus-ORF1 and 97.2% to the closest Hu/GII/ITA/2013/GII.4_Sydney2012/PA13 strain (KF378731) in the ORF2-3′ terminus. The recombination breakpoint was predicted to be at nucleotide position 5088, which is in a sequence between ORF1 and ORF2 that is conserved on norovirus genomes (7).

To our knowledge, this is the first report of a complete genome sequence of norovirus GII.P16-GII.4_Sydney2012. Norovirus infection is caused mostly by the GII.4 genotype, followed by GII.2, GII.3, GII.6, and GII.17 (8–11). The most prevalent GII.4 contained GII.P4 and GII.Pe RdRp sequences, and GII.P16 strains with various VP1 genotypes have been detected in some countries (12–20). The amino acid identity was 95 to 100% among GII.P16 RdRp sequences, and these diverged phylogenetically into two clusters. GII.P16 showed an amino acid identity of 88 to 93% to GII.P4 and GII.Pe in the RdRp sequences. Some proteins within ORF1 are associated with the pathogenesis of norovirus (21, 22). Additionally, recent reports have shown that an interaction between the human norovirus GII RdRp and GII VP1 proteins, but not the murine norovirus VP1, enhanced host RIG-I-dependent interferon signaling activity via replicative RNA in a human cell line, whereas the GII VP2 downregulated the innate immune signaling (23, 24). Norovirus GII exhibits genetic diversity among the genotypes in the capsid sequence, which is crucial for host entry and the production of blocking antibodies, and also in the nonstructural polyprotein-coding sequence (25–28). Therefore, appropriate combinations of ORF1 and ORF2-ORF3 sequences may be associated with the enhancement of norovirus pathogenesis and infectivity, which leads to the prevalence of restricted genotypes.

### Accession number(s).

The GenBank accession number for the norovirus Hu/GII/JP/2016/GII.P16-GII.4_Sydney2012/Kawasaki194 genome sequence is LC175468.
